# Identifying patterns of co-occurring chronic conditions preceding dementia: An unsupervised machine learning approach using health administrative data

**DOI:** 10.23889/ijpds.v9i5.2849

**Published:** 2024-09-18

**Authors:** Laura C. Maclagan, Daniel A. Harris, Xuesong Wang, Mohamed Abdalla, Tomi Odugbemi, Ruth Ann Marrie, Peter C. Austin, Richard H. Swartz, Sandra E. Black, Myuri Ruthirakuhan, Colleen J. Maxwell, Susan E. Bronskill

**Affiliations:** 1 ICES; 2 Department of Health Services, Policy & Practice, Brown University School of Public Health; 3 Institute for Better Health, Trillium Health Partners; 4 Departments of Internal Medicine and Community Health Sciences, Max Rady College of Medicine, Rady Faculty of Health Sciences, University of Manitoba; 5 Institute of Health Policy, Management & Evaluation, Dalla Lana School of Public Health, University of Toronto; 6 Department of Medicine (Neurology), Hurvitz Brain Sciences Program, Sunnybrook Health Sciences Centre, University of Toronto; 7 Schools of Pharmacy and Public Health Sciences University of Waterloo

## Objective

Individual risk factors for dementia are well known, but the influence of co-occurring chronic conditions has not been considered. We identified clusters of chronic conditions using an unsupervised machine learning approach and examined associations with incident dementia.

## Approach

Using linked population-based administrative databases, we followed all community-dwelling adults aged 40-54 years in Ontario, Canada from April 2002 until March 2019 for incident dementia. We estimated the prevalence of 29 chronic conditions using validated algorithms and/or diagnosis codes. We reduced dataset dimensionality using multiple correspondence analysis and a fuzzy c-means clustering algorithm identified the optimal number of clusters (between 3-6 tested). Associations between clusters and incident dementia were examined using a cause-specific hazard model adjusted for sociodemographic characteristics and accounting for the competing risk of death.

## Results

We identified 82,359 eligible individuals (random 3% sample of total eligible individuals; mean age 46.5 years; 50.4% female). Regression analyses were based on 5 comorbidity clusters (fuzzy silhouette index:0.69). Compared to the low comorbidity cluster, persons in the cerebrovascular disease/metabolic (HRadj=3.06, 95%CI[2.42,3.86]) and neuro-related/mental health clusters (HRadj=2.51, 95%CI[2.05,3.07]) had the highest rates of incident dementia, followed by the cardiovascular risk factor cluster (HRadj=1.66,95%CI[1.32,2.09]). Persons in the cancer cluster did not have an increased incidence of dementia (HRadj=0.96,95%CI[0.77,1.20]).

## Conclusions

We found significant associations between machine learning-derived clusters of chronic conditions and dementia.

## Implications

Unsupervised machine learning approaches to identify clusters of chronic conditions may be a useful tool for considering the impact of multimorbidity on dementia risk.

